# Atlanta Society of Medicine

**Published:** 1899-04

**Authors:** 


### SOCIETY REPORTS.

### ATLANTA SOCIETY OF MEDICINE.

*Meeting March 16.*

Dr. Wolff read a paper upon "Protection Against Fraudulent Malpractice Suits."

DISCUSSION.

*Dr. C. D. Hurt:* I think Dr. Wolff has covered the ground so well that there is not so much left to be said. It is very true that the medical profession is suffering materially from these malpractice suits, and they seem to grow more numerous and annoying. Just how to arrest them is the question, but the medical profession should take some action to protect itself in this city, as well as in the entire State. Our tendency is to take the best possible care of our patients, and I do not know a man in the profession who neglects his patients. The charge against a man as being negligent, when he has done his best, should not be supported, and he should not be sued. Dr. Wolff brought out some important points. I do not believe that the profession is entirely clear of some error in this matter. Some time we talk indiscreetly among ourselves, or about ourselves in the presence of laymen, and this gives them an idea that we are not exactly in accordance with one another. Such a small matter as a little shrug of the shoulders, etc., is calculated to increase these suits.

Dr. Wolff's point about a physician having around him a third person in giving an anesthetic is well taken, although I do not know of any man who gives an anesthetic alone. Exaggerated statements are very common in regard to medical matters, and we have some very ludicrous statements in the newspapers; and, by the way, it seems to me that the newspapers have, especially of late, developed a sensational fever as to medical affairs. I noticed in to-day's the announcement in flaring head-lines that a man was found to be insane after he had been hung. Something should be done to prevent all such statements being made, as they are detrimental to the profession. We should have committees all over the State to keep a watch on these things, and keep them down by denouncing the editors and reporters. I believe much can be accomplished by concerted action, and we cannot do much unless we have this.

*Dr. Hutchins:* I have not had much experience with damage suits. Some years ago, while trying to collect a bill, one lady said that I had put lead in a prescription and that she would bring suit. I requested her to pay the bill and then bring suit. The account was paid, but no suit was brought.

*Dr. Duncan:* I believe nearly every individual who intends bringing suit, unless it is blackmail, will consult some physician before doing so, and they will try to get some doctor friend to advise them to bring suit. I always discourage any such persons. If their doctor will discourage such things, most suits will not mature. In blackmail cases a physician will hardly be consulted.

*Dr. Hardon:* I think that Dr. Hurt and Dr. Duncan touched upon one of the most important points of this subject. It is not only true that in most cases there is a doctor behind the plaintiff, but the doctors also go on the stand and testify for them. There is a case in court right now in which one member of this Society is being sued, and the other side has another member of this Society to testify. He is ashamed to go on the stand, but his testimony is being used. No lawyer would take a case if he could not get a doctor to testify for him. Therefore we should take some action and discourage physicians appearing as witnesses for the plaintiff in suits of this kind.

*Dr. Childs:* We have professional witnesses in the medical profession just as they have "snide" lawyers. We have men who make more money testifying than by their practice. Like Dr. Hardon, I believe in discouraging all such. I remember that the late Dr. N. O. Harris was sent for by an old negro to vaccinate a little child and they claimed that he used pus from another case, when in reality he used the bovine virus. The arm was very bad and the negro brought suit. Dr. Harris had me go out and examine the child and I found mucous patches about the anus and in other parts of the body. The child had the syphilitic teeth, etc., and the evidence showed it to be a pure case of hereditary syphilis. Dr. Harris died and the suit never came up, and it would have fallen through anyway. In regard to these cases, I believe that it is very unethical for a man to testify against another. As Dr. Hurt said, men are not often negligent of their duty; some may be through ignorance, but we have a State Board and it is their duty to find out if a man is not able to practice. Of course, if a man should be neglectful, there is some ground for a malpractice suit; but when the law examines a man and gives him license to practice it should give him protection.

*Dr. Olmsted:* I do not know that I can add anything to the excellent discussion and paper by Dr. Wolff. I think the main part of the discussion puts the matter where it should be, that is, at home. These cases would all be lost if every member of the profession did his duty in regard to them. The English language fails me to express my opinion of such a man who will testify against another. There are some cases of gross neglect, but they do not come under this head, for the majority of cases are of the fraudulent variety. As has been mentioned, these cases are always aided and abetted by a medical man, and I think it is our duty when one is attacked to stand by him and go to court and testify, and make it a personal matter to see that a reputable brother is indorsed and supported, rather than they should be brought in and made to feel embarrassed by some absurd hypothetical case which the lawyer may mix up with the present case. I understand that some of these cases are coming up in the spring courts, and I certainly do not envy the gentlemen (whom I do not know) who would give evidence against a brother physician in the case which has been referred to by Dr. Hardon. The names do not concern us here, it is the principle; when we get to discussing these matters we lay aside the personal. We should remember that any of us are liable to be put in the same embarrassing position, and should feel that we are among brothers who are willing to stand up for us in these suits, which are an abomination to all concerned.

*Dr. Richardson:* I have nothing to say, except to compliment the gentleman who read the paper and to indorse the sentiment which has been so well expressed. A remedy has been suggested by Dr. Olmsted and others, and that is, union and organization. As regards the merits and ability of the men being sued, why such men as Flint, Loomis, etc., they all make mistakes, and regardless of mistakes, if a man has done his best, he should be indorsed.

*Dr. Wolff* (closing the discussion): I purposely omitted the reference to any doctors, as I referred more to the foes from without, and I thank the gentlemen for the discussion of the paper.

Dr. Thos. H. Hancock read a paper on "Circumcision."

DISCUSSION.

*Dr. Duncan:* All circumcisions I have done, or seen done, removed the entire prepuce "at one sitting." I have witnessed a number of the Jewish circumcisions and he describes this very accurately, but if I was going to operate upon a child or adult, I would finish up the job at once. I think the operation of circumcision is a good thing, and if the Gentiles would follow the course of the Jews, they would be benefited by it. In doing the operation I do not use an anesthetic. Recently I operated upon two small children in the same family. I circumcised one and turned him loose and he ran about and watched me operate upon the other one and seemed to enjoy it. Circumcision is very simple, and I do not see any necessity for making two operations.

*Dr. Parks:* I have been very much interested in Dr. Hancock's paper. I took the ground before the American Medical Association that in little children circumcision is unnecessary. We wonder how children can be born perfect in everything except the prepuce. The reason, I think, is that at the junction of the mucous membrane and the skin there is a little constricted band, and it sometimes undergoes inflammation, and this band by adhesion binds down the prepuce. If these adhesions are broken up and then gradually dilate the prepuce by ballooning every other day until the constricted band is broken up, the prepuce can then be retracted, and it will gradually shorten. I have demonstrated this in over 200 cases. I discovered this in treating a case of simple urethritis in a young man. He went off on a vacation and when he returned the inflammation had extended to the prepuce, and where before one-third of the glans was exposed, the prepuce now extended half an inch beyond the glans. After the inflammation was reduced I broke up this band and the prepuce gradually retracted and showed two-thirds of the glans. I do not believe in circumcision. I will not say that it is malpractice, but I do think it is mutilation.

*Dr. Childs:* I cannot understand why Dr. Hancock does the operation in sections. The complete operation is very simple. I was operated upon after I was grown, and I did not have to stay away from meals, nor did it give any serious inconvenience. Not long since I operated upon a man forty years of age, and it did not give him any trouble and did not keep him awake at night. Dr. Champion and I do a number of operations and we have no trouble, and I cannot understand why he would do the operation as he mentions. I think every male child should be circumcised. They are not as liable to contract diseases. I have had a great deal of experience, and I am sure that urethritis responds more quickly in cases that have no foreskin. The foreskin often retains the discharge, and this retained pus often reinfects the case. I have never seen more than one case of syphilis in a Jew. They do not have it because they have no foreskin to retain the poison, and there is their advantage. In children the operation is no trouble, and it often relieves nervous conditions. I know in the case of my own child, which was operated upon eight weeks ago, it was restless and irritable before the operation, and the entire prepuce was found to be adherent, and since then the child rests better and is improving since the operation.

*Dr. G. P. Robinson:* I think the old Jewish custom for children is a good one. As Dr. Parks says, there are many cases in which you can break up the adhesions and dilate the prepuce and it answers very well, but still it does not take the place of circumcision. The child for a number of years is dependent upon the mother or nurse, and they do not as a rule draw back the skin and keep the parts as clean as should be. As we know, there is a chain of nervous symptoms in a large number, which are due to nothing more than the irritation of the accumulations of smegma under the foreskin. Where the operation is done the parts are easily kept clean, and in the majority of cases they are a great deal better. In my own child there was a chain of nervous symptoms which disappeared after the operation, and also there is less liability to disease as they grow older.

*Dr. Champion:* As to the method of doing the operation, I use the clamp. I inject the cocaine between the mucous membrane and the skin, and then put on the clamp, remove the prepuce and then suture the mucous membrane and the skin, using continuous silk sutures. After five days we can take the sutures out, and after eight days remove all dressings and it is healed completely. I do not see any reason why the operation should be done in sections, when the entire operation can be done at one time and the patient be practically well within five days. I enjoyed the paper very much, and especially the history of the operation.

*Dr. Olmsted:* I can hardly see the advantage of operating in sections, and I should be afraid that some would not come back to have the other side cut off. As to the manner of operating, I have preferred to make the dorsal incision and take off the flaps right and left. I have seen some cases which have had trouble with erections. In regard to children, I must confess that the majority are much better after being circumcised. I have had a number of cases which were very annoying with nervous symptoms, such as reflex paralysis, etc., and which were relieved by operation. In some cases in which there were adhesions I have broken them up by ballooning up the prepuce, but as a rule there are dense adhesions which can only be relieved by operation. In regard to phimosis, it also causes irritation. I saw a case in a little child six years old, and the little fellow would handle his penis and there would be an erection, and he evidently enjoyed manipulating it. It is very desirable to remove all that tendency, for it will give him enough trouble later on. If the other treatment is instituted and it is washed every day, it brings his attention to the part, which is just what you want most to avoid. I do not think it should be left to the mother to take care of this, and I am in favor of circumcising infants.

*Dr. Divine:* This subject seems to be thoroughly understood, but like the others, would like to know why the doctor performs the operation as he does.

I would like to know what experience the gentlemen have had in circumcising the clitoris to break up nervous attacks, or whether they can be remedied by this means, and whether they have done this?"

*Dr. Richardson:* I have had no experience with the clitoris and know nothing about it.

In 1870 Dr. Louis Sayre demonstrated that the prepuce in children produced paralysis, curvature of the spine, chorea, and various reflex phenomena, and we know it produces inflammation, urethritis, and balanitis, and one phase which has not been mentioned, that the operation prevents masturbation, and I am in accordance with the gentlemen in the statement that it should be practiced in every child. The operation is very simple, and I do not see any necessity for ballooning or doing the operation in sections. I usually use elastic sutures and dress it, and this ends it. If it is aseptic, you can turn it loose in a week.

*Dr. Hardon:* I do not know that there are any recorded cases in which circumcision has been done in female children, and I do not know of any case reported of a train of nervous symptoms which were produced by similar conditions in the female. But there is a class of cases in nervous adult women where such symptoms are produced by adhesions of the prepuce of the clitoris, and this is very similar to the male. The operation has frequently been done of breaking up the adhesions and removing the accumulations of smegma with good results. Of course, the analogy in the two makes it easy to understand why such operations are good, and while circumcision has not been extensively advocated in females, yet it has been done for the reason stated. Of course, the extent of the adhesions are much less than in the male, as the small size of the prepuce of the clitoris makes it a necessity. This is all that I have to say in answer to the question as to the operation in females.

*Dr. Childs:* I wish to mention briefly a case which was relieved by operation. About seven years ago a young man, who is now a member of this Society, was being treated by me for simple urethritis, and one day I was talking with him about chronic salivation and he told me that he had been troubled with it for some time. I put him on belladonna and other things, but got no result. I had read a report somewhere which stated that this had been relieved by circumcision. I suggested circumcision to him, as he needed it, and after operating the condition was relieved.

*Dr. Goldsmith:* I must differ with Dr. Hancock in the technique of the operation. I agree with Dr. Olmsted in the statement that I would be afraid the patient would not come back to have the other side removed. It is not necessary where the operation can be finished at once. In my cases I use simply a narrow band of sterile gauze over the incision, and over this a strip of plaster, applying it some little distance up the body of the organ. In this way the strips form a splint to the parts, and properly applied, we have very little swelling. Of course, I have the patient to come back next day if there is any swelling, and I dress it again and this ends the condition. In the majority of cases my patients suffer with erections, and I usually give them a tablet of morphine to take. In children I believe in circumcising every male child. We find in some children that the smegma has collected under the prepuce and become so hard as to produce great irritation. A very small amount of smegma will of itself cause irritation, and this increases the longer it remains. As has been mentioned, numerous nervous symptoms follow these cases. The Jew is an evidence of the value of circumcision. I have never seen a case of syphilis in a Hebrew, and circumcision has certainly not produced any deterioration in the size of the organ, for I have always found it up to the standard.

*Dr. Hancock:* The chief purpose of this paper was to bring out the importance of the two operations in circumcisions. I found for a long time that many of the patients had considerable swelling, and all had erections at night, which causes considerable pain. I remember one patient who would beat his knees with a walking stick to reduce it. No one can dispute the fact that there is considerable pain after the operation. It is very little more trouble to do the operation in two sections, and I find that there is not one-fifth the pain as when the complete operation is made.

In regard to sutures, of course, we would not think of using a continuous suture, either silk or catgut, because, when the erection comes on, such a suture will produce strangulation and cause intense pain.

In regard to the circumcision of females, I find that in some book it is mentioned that the Pesians and Egyptians practice regularly the circumcision of their females as well as their males.

REPORT OF CASES.

*Dr. Wolff:* I have a very interesting case to show the Society. This unfortunate being has been reduced to a tramp on account of his condition, as he is a hermaphrodite. I have never seen a case so well marked before. This person's means of livelihood consists in exhibiting himself for physicians. He is thirty years of age, a native of Germany, and up to the time of twelve years of age wore female attire and since then, for some reason which I did not learn, has been wearing male attire. This person is somewhat feminine in appearance, has large hips, has breasts and is beardless, and has no axillary hair and the hair on the pubes grows in a triangular shape as in women. However, the hands are masculine, and also the feet. As to hermaphrodism, it is divided into two varieties, hermaphrodismus verus and pseudohermaphrodismus. In the true variety there are both ovaries and testicles, with the function of both, and the person can play either the masculine or feminine rôle during copulation. The false variety is not very infrequent, and this is again divided into two varieties, depending upon whether it resembles the male or the female. I think this is a male with a very bad case of cleft scrotum and hypospadias. You will notice just below the mons veneris there are two folds which resemble the labia majora and each one contains a mass which is the size, shape, and consistency of a testicle, and between these there is what seems to be a rudimentary penis but which is imperforate.

*Dr. Stephens:* I would like to ask whether any gentleman can distinguish between the epidemic of measles we are having now and that of three or four years ago? As you know, measles do not usually recur, but this seems to be the case with these we are having now.

*Dr. Duncan:* I have seen those cases that Dr. Stephens speaks of and I have called them rubella, or rotheln. There is this peculiarity, there will be a little fever and slight catarrhal symptoms which will be observed during to-day and to-morrow, and the next day will have an eruption, that is, coming out on the second day. Some do not complain at all until the eruption is discovered, and as a rule, they do not go to the fourth day. I have not seen a case this winter which I considered genuine measles. One case of a little girl had an eruption when I first saw her and had not complained until the day before. A few days later her brother was attacked and broke out on the second day. His case was a little more severe, but neither were given any treatment and they got well in a few days and much sooner than measles.

